# Stylet-protected guidewire-exchange for infected tunneled hemodialysis catheters: Outcomes from a single-center retrospective study

**DOI:** 10.1177/11297298261452710

**Published:** 2026-07-07

**Authors:** Elettra Lomeo, Francesca Maria D’Ascenzo, Antonio Sturniolo, Roberto Mangiacapra, Fabio Aureli, Francesco Pesce, Giuseppe Grandaliano

**Affiliations:** 1 Nephrology, Dialysis and Transplantation Unit, Department of Medical and Surgical Sciences, Fondazione Policlinico Universitario A. Gemelli IRCCS, Rome, Italy; 2 Department of Translational Medicine and Surgery, Universita’ Cattolica del Sacro Cuore, Rome, Italy; 3 Division of Renal Medicine, Ospedale Isola Tiberina-Gemelli Isola, Rome, Italy

**Keywords:** CRBSI, CVC, guidewire-exchange, vascular access preservation, CVC replacement

## Abstract

**Background:**

Catheter-related bloodstream infections (CRBSIs) are the main complications associated with the use of permanent central venous catheters (CVCs). However, standardized management protocols for infected tunneled CVCs remain heterogeneous. We present an innovative stylet-protected guidewire-exchange technique designed to minimize contamination and preserve vascular access sites.

**Methods:**

We performed a retrospective, observational, single-center study of infected tunneled CVC replacement at the Nephrology Unit of Policlinico A. Gemelli Foundation (Rome) from January 2019 to June 2024. Follow-up ranged from 1 to 4 years. The primary endpoint was infection-free survival, defined as time to relapse (recurrent CRBSI with the same pathogen) or reinfection (a different pathogen).

**Results:**

A total of 99 CVC exchanges were performed in 99 patients (M 50.5%, F 49.5%; median age: 72 years, IQR 62–78.5). To assess the safety of the technique, guidewire cultures were obtained, confirming persistence of the initial CRBSI pathogen. Despite positive guidewire cultures, relapse by the same pathogen occurred in only 2% of cases: one *Pseudomonas aeruginosa* (at 3 months) and one *Staphylococcus aureus* (at 8 months). Reinfection with different pathogens occurred in 11.1% of cases (11 patients) after a median of 283 days (IQR 121–642). All catheters maintained extracorporeal blood flow ⩾250 mL/min, ensuring adequate dialysis.

**Conclusions:**

This stylet-protected guidewire-exchange demonstrated excellent infection-free survival, including infections caused by high-risk pathogens such as *Staphylococcus aureus*. These results support guidewire exchange as a safe, effective and reproducible method for replacing infected tunneled CVCs in hemodialysis patients.

## Background

Reliable vascular access is essential for patients with end-stage kidney disease (ESKD) undergoing hemodialysis (HD). Despite the 2019 KDOQI guidelines promoting “right access for the right patient,”^1^ more than 80% of incident hemodialysis patients in the United States still initiate treatment with a central venous catheter (CVC) due to advanced age, exhausted vascular access, comorbidities, or late nephrology referral.^2–4^

However, increasing CVC use has led to a rise in complications, particularly catheter-related bloodstream infections (CRBSIs), which represent a major cause of morbidity, mortality, and hospitalization among HD patients.^1,5^ CRBSIs account for one to five episodes per 1000 catheter-days and are most frequently caused by Gram-positive organisms, mainly *Staphylococcus aureus* and coagulase-negative staphylococci.^6–10^ Infections by these pathogens often lead to severe complications such as endocarditis and osteomyelitis.^7^ While current guidelines recommend catheter removal in cases of severe sepsis or infection with high-risk organisms, repeated catheter removals and reinsertions may exhaust available vascular access sites, leading to stenosis, thrombosis, or complete loss of venous access.^8,11^

In hemodialysis patients, preserving venous access is critical, particularly when vascular sites are limited. Catheter removal is indicated in cases of severe sepsis with hemodynamic compromise or metastatic infection (endocarditis, septic arthritis, or others),^1,6^ whereas other cases might be managed with guidewire exchange to minimize vascular injury. CVC-guidewire exchange offers a solution but is controversial due to the risk of reintroducing infection via the contaminated subcutaneous tract. Although some evidence suggests comparable infection-free survival between guidewire exchange and catheter removal,^11–14^ apprehension about reintroducing infection via the contaminated tract and the limited availability of detailed, standardized procedural protocols have restricted its clinical uptake.

For these reasons, we evaluated a stylet-protected guidewire-exchange technique specifically designed to minimize contamination risk and preserve vascular access in patients with CRBSI.

## Patients and methods

### Study design and data collection

We conducted a retrospective, observational, single-center study of tunneled CVC exchanges performed using the stylet-protected guidewire-assisted technique by nephrologists at the Nephrology Unit of Fondazione Policlinico Universitario A. Gemelli IRCCS, Rome, Italy, between January 2019 and June 2024. Clinical data were retrieved from electronic medical records, including demographic, clinical, microbiological, and procedural information. Collected variables included age, sex, CVC dwell time and insertion site, dialysis vintage, etiology of kidney disease, presence of a pacemaker (PMK) or endocarditis, catheter-tip thrombosis, serum albumin, and antibiotic therapy.

The primary outcome was infection-free survival, defined as freedom from relapse (recurrent CRBSI caused by the same pathogen) or reinfection (CRBSI caused by a different pathogen), together with clinical resolution of CRBSI. Clinical resolution was defined as the absence of symptoms (fever, chills, or other systemic infection symptoms), normalization of inflammatory markers (white blood cell count, C-reactive protein, or procalcitonin), and negative follow-up blood cultures.

Secondary outcomes included adequate catheter function, defined as extracorporeal blood flow ⩾250 mL/min during dialysis, and all-cause mortality.

### Study population

Eligible patients were adults (⩾18 years) receiving maintenance hemodialysis at dialysis centers affiliated with our institution, with microbiologically confirmed tunneled CVC-related bloodstream infections, defined by positive blood cultures and a differential time to positivity (DTP) ⩾2 h between catheter-drawn and peripheral blood cultures, with or without associated exit-site or tunnel infection. All study participants were catheter-dependent at the time of diagnosis and did not have a functioning arteriovenous fistula due to exhausted vascular access, unfavorable vascular anatomy, or significant comorbidities.

Exclusion criteria were: age <18 years, suspected but microbiologically unconfirmed CRBSI, and suspected tunnel or exit-site infection without microbiological confirmation.

All procedures were performed within 48 h of CRBSI confirmation. All patients with *Staphylococcus aureus* bacteremia (and other typical endocarditis-associated pathogens) underwent systematic echocardiographic evaluation. Endocarditis was diagnosed according to Duke criteria. Post-procedural follow-up ranged from 1 to 4 years (mean 2 years), including echocardiography, inflammatory markers (C-reactive protein, procalcitonin, white blood cell count), and repeat blood cultures (peripheral and catheter-drawn).

### Statistical analysis

Data were analyzed using IBM SPSS Statistics version 29.0 (IBM Corp., Armonk, NY, USA). Continuous variables are reported as medians with interquartile ranges (IQRs) or means ± standard deviations (SD), as appropriate. Categorical variables are expressed as counts and percentages. Infection-free survival after guidewire exchange was estimated by the Kaplan–Meier method, defining the event as relapse or reinfection by any pathogen and censoring at death or last available follow-up.

Technical success and complication rates were expressed as proportions with 95% confidence intervals (CI) calculated using the Clopper–Pearson exact method. Because of the descriptive and exploratory nature of this study, no formal hypothesis testing or multivariable analysis was performed.

### Procedure technique

The technique was applied to all CRBSI cases, regardless of pathogen type. Both jugular and femoral tunneled CVCs were eligible. Significant alterations in inflammatory markers (such as C-reactive protein or procalcitonin levels) or the presence of endocarditis were not considered contraindications. Tunnel infection of the CVC did not preclude the procedure, provided that adequate space was available to create a new tunnel on the same side. The only contraindication was hemodynamic instability requiring immediate catheter removal for patient stabilization. All procedures were performed under local anesthesia and had an average duration of ~30 min.

Patients fasted for at least 6 h prior to the intervention, in case procedural sedation was required. Pre-procedural assessment included laboratory testing (complete blood count, prothrombin time and partial thromboplastin time) as well as a standard electrocardiogram. All procedures were performed under strict aseptic conditions in a fluoroscopy-equipped interventional nephrology suite. All patients received a single dose of intravenous antibiotic therapy prior to the procedure.

Before entering the procedure room, the lock solution was aspirated from both catheter lumens and any anchoring sutures were removed. Once the patient was positioned on the operating table, the catheter was clamped ~0.5 cm distal to the exit site and the external portion of the catheter was cut away (Figure 1(a)). After skin disinfection, a sterile field was prepared. Following local anesthesia, the CVC was isolated at its nearest point of venous entry, double clamped and cut downstream from the clamps (Figure 1(b)).

**Figure 1.**
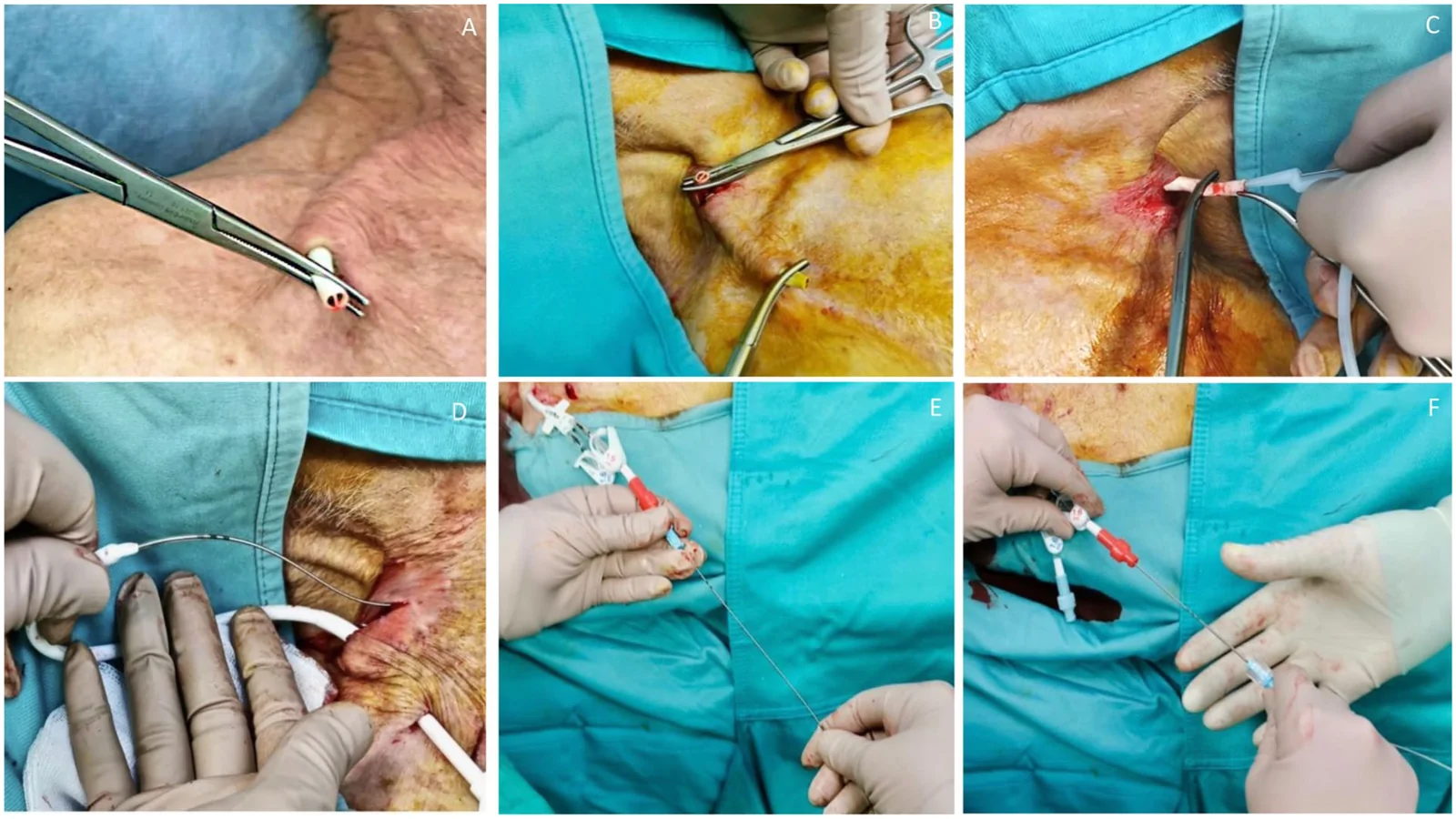
Steps of the guidewire-assisted exchange of a tunneled CVC: (a) external catheter branches are transected near the skin exit site, (b) the catheter is exposed at the venous entry site and clamped, (c) a J-tip guidewire is introduced through one lumen, and the old catheter is withdrawn under fluoroscopy, (d) a new subcutaneous tunnel is created; the new CVC is advanced with the guidewire positioned inside the internal stylet to avoid luminal contamination, and (e, f) the stylet-guidewire assembly is withdrawn as a single unit, and final positioning is confirmed.

The cuff was then exposed and detached from the surrounding tissue, allowing removal of the external limb of the CVC. All surgical instruments used during this step were subsequently removed from the sterile field. One of the two lumens near the neck was clipped and a metal J-tip guidewire was inserted through the remaining free lumen (Figure 1(c)).

The proximal part of the catheter was unclamped and carefully withdrawn, while ensuring that the guidewire remained at the correct depth, as confirmed by fluoroscopy (typically 1.5–2 vertebral bodies below the carina). Catheter placement was simulated by measuring the guidewire length from the jugular to the J-tip to determine the appropriate exit site. A new anterograde subcutaneous tunnel was created. The new CVC, pre-loaded with its internal stiffening stylet, was advanced over the guidewire. Crucially, the guidewire was threaded through the hollow lumen of the internal stylet. This configuration ensures that the potentially contaminated guidewire did not come into direct contact with the luminal surface of the new catheter (Figure 1(d)). Once the catheter was correctly positioned, the guidewire and stylet were removed simultaneously as a single unit (Figure 1(e) and (f)), leaving the sterile catheter lumen untouched.

A fluoroscopic inspection was then performed to confirm proper CVC tip positioning. The procedure was completed by suturing the skin and fixing the CVC to the skin with non-absorbable monofilament sutures. After the procedure, catheters lumens were locked according to standard institutional protocols (taurolidine/heparin/citrate as the preferred antimicrobial lock; alternative 4% sodium citrate or unfractionated heparin).

All procedures were performed by experienced interventional nephrologists. Real-time fluoroscopy is essential to minimize complications and confirm accurate tip positioning.

In rare cases of older, ingrown tunneled catheters, interventional radiology support may be advisable. When needed, catheter removal can be facilitated using sequential endoluminal balloon dilatation under fluoroscopic guidance to disrupt fibrotic adhesions and allow controlled extraction.

## Results

During the study period, 99 tunneled CVC guidewire exchanges were performed in 99 patients (50.5% male and 49.5% female; median age: 72 years, IQR 62–78.5). Table 1 summarizes baseline demographic and clinical characteristics. Primary kidney disease and common comorbidities included diabetes mellitus (33.3%), glomerulonephritis (12%), coronary artery disease (32%), peripheral vascular disease (19%), autosomal dominant polycystic kidney disease (8%), history of kidney transplantation (13%), and immunosuppression (18%).

**Table 1.** Baseline demographic and clinical characteristics of patients with CRBSIs.

Characteristic	Value
Demographics	
Gender, male, *n* (%)	50 (50.5%)
Age, median (IQR), years	72 (62–78.5)
Dialysis history	
Dialysis vintage, median (IQR), months	36 (7.5–60)
Catheter dwell time, median (IQR), months	30 (25–34)
Comorbidities, *n* (%)	
Diabetes mellitus	33 (33.3%)
Coronary artery disease	32 (32%)
Peripheral vascular disease	19 (19%)
Immunosuppression	18 (18%)
History of kidney transplantation	13 (13%)
Glomerulonephritis	12 (12%)
ADPKD	8 (8%)
Clinical status at exchange, *n* (%)	
Presence of pacemaker	7 (7%)
Endocarditis	6 (6%)
Catheter-tip thrombosis	4 (4%)
Serum albumin <25 g/L	23 (23%)

IQR: interquartile range.

Baseline demographic and clinical characteristics of patients undergoing stylet-protected guidewire-exchange of tunneled CVCs. Data are presented as absolute numbers or percentages, unless otherwise specified. Median age is reported with IQR.

The median dialysis vintage was 36 months (IQR 7.5–60), and the median catheter dwell time was 30 months (IQR 25–34). A pacemaker was present in 7% of patients; endocarditis (diagnosed according to the Duke criteria) and catheter-tip thrombosis occurred in 6% and 4% of cases, respectively. Serum albumin levels at CRBSI onset were <25 g/L in 23% of patients, with a median of 30 g/L (IQR 25–34).

Most CVCs (84%) were placed in the right internal jugular vein. Among Gram-positive pathogens, *Staphylococcus aureus* was predominant (73.2%), followed by coagulase-negative staphylococci (CoNS; 19.6%; Figure 2(a) and (b)). Guidewire cultures were obtained, confirming persistence of the initial CRBSI pathogen.

**Figure 2.**
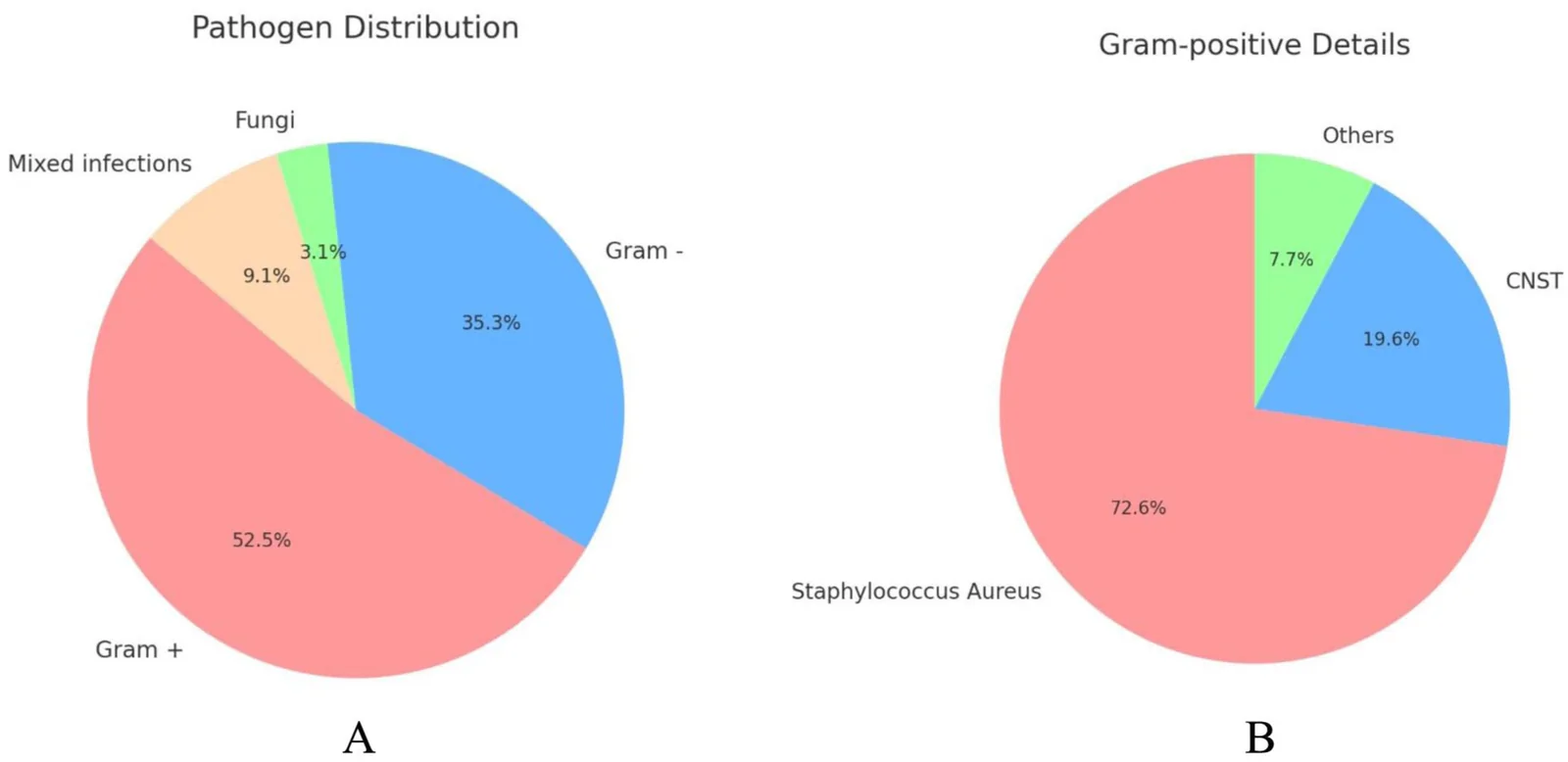
Pathogen distribution and Gram-positive isolate characteristics in patients with CRBSIs: (a) pathogen distribution: Gram-positive 52.5%, Gram-negative 35.3%, mixed 9.1%, fungi 3.1% and (b) Gram-positive isolates: 73% *S. aureus*, 20% coagulase-negative staphylococci, 7% others. CRBSIs: catheter-related bloodstream infections.

At the time of the procedure, antibiotic management was heterogeneous, reflecting real-world clinical practice. Overall, 80% of patients received intravenous antibiotic therapy prior to the procedure. Among these, 31 patients initiated empirical antibiotic treatment (vancomycin, cefazolin, or piperacillin/tazobactam) which was subsequently adjusted according to antibiogram results; whereas in 48 patients pathogen-directed antibiotic therapy was started based on microbiological data. The remaining 20% of patients presented upon hospital admission without ongoing antibiotic therapy and initiated intravenous antibiotics therapy on the day of the procedure, administered at least 1 h before catheter exchange. The duration of systemic antibiotic therapy was variable, with a maximum of 14 days, and was tailored according to pathogen susceptibility and clinical response. Overall, 14 patients (14.1%) received antibiotic therapy for <5 days, 45 patients (45.5%) received treatment for 5–10 days, and 40 patients (40.4%) received therapy for more than 10 days, with a maximum duration of 14 days.

The relapse rate was 2% (95% CI 0.2–7.0). One case occurred within 3 months and was associated with endocarditis caused by the same pathogen *Pseudomonas aeruginosa*; this patient had received 7 days of systematic antibiotic therapy. The second case occurred 8 months after the initial infection and involved *Staphylococcus aureus*; this patient had received 10 days of antibiotic therapy (Figure 3(a)).

**Figure 3.**
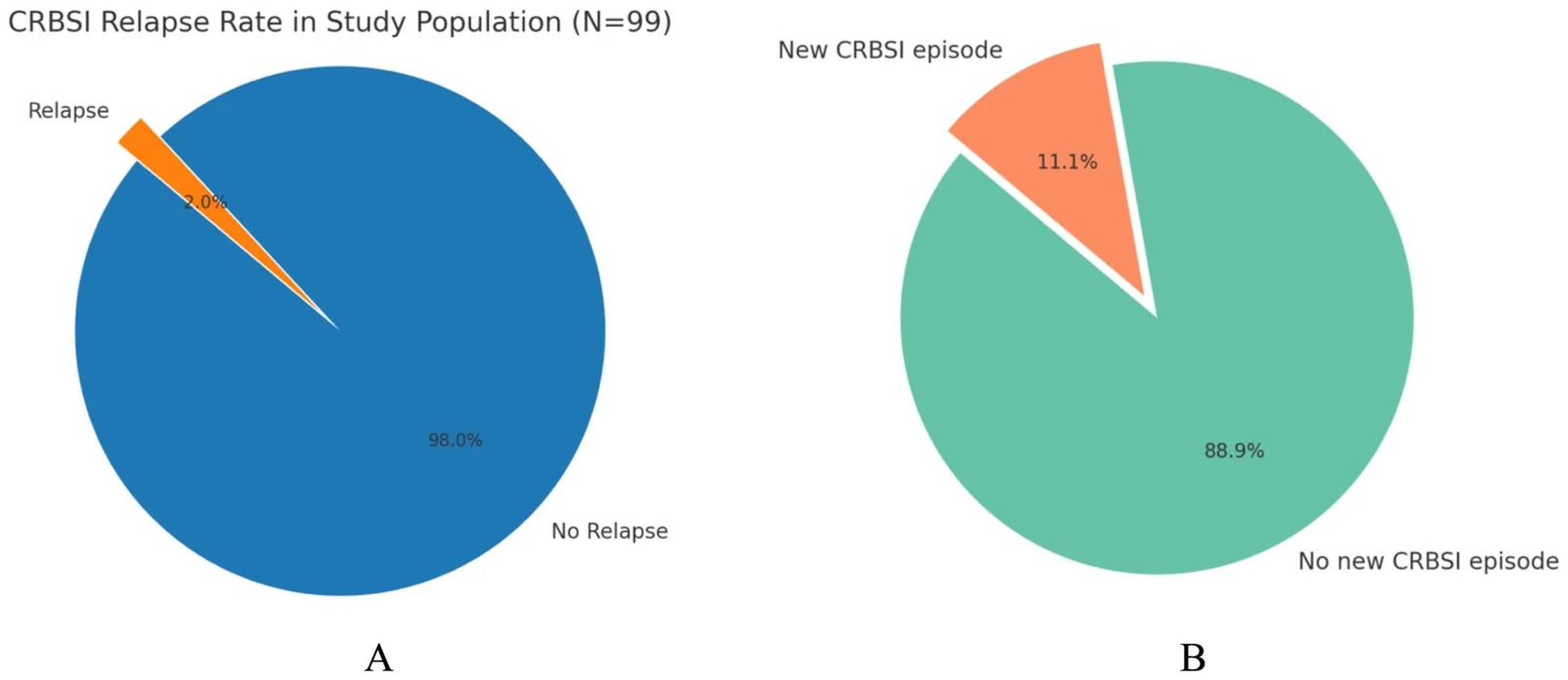
Relapse and reinfection rates following guidewire-assisted tunneled CVC exchange: (a) relapse with the same pathogen occurred in 2% and (b) the reinfection rate with a different organism was 11.1%.

Reinfection with different pathogens occurred in 11.1% (95% CI 5.7–19.0) of patients after a median of 283 days (IQR 121–642; Figure 3(b)). Kaplan–Meier analysis demonstrated sustained infection-free outcomes, with estimated infection-free survival rates of 93.7% at 12 months, 87.3% at 24 months, and 84.3% at 36 months (Figure 4). During the follow-up period, overall mortality from unrelated causes was 14.1%: eight of the 14 deaths occurred in patients older than 80 years. Among patients aged >80 years (*n* = 21), survival during follow-up was 61.9%; deaths were unrelated to CRBSI or CVC exchange procedure.

**Figure 4.**
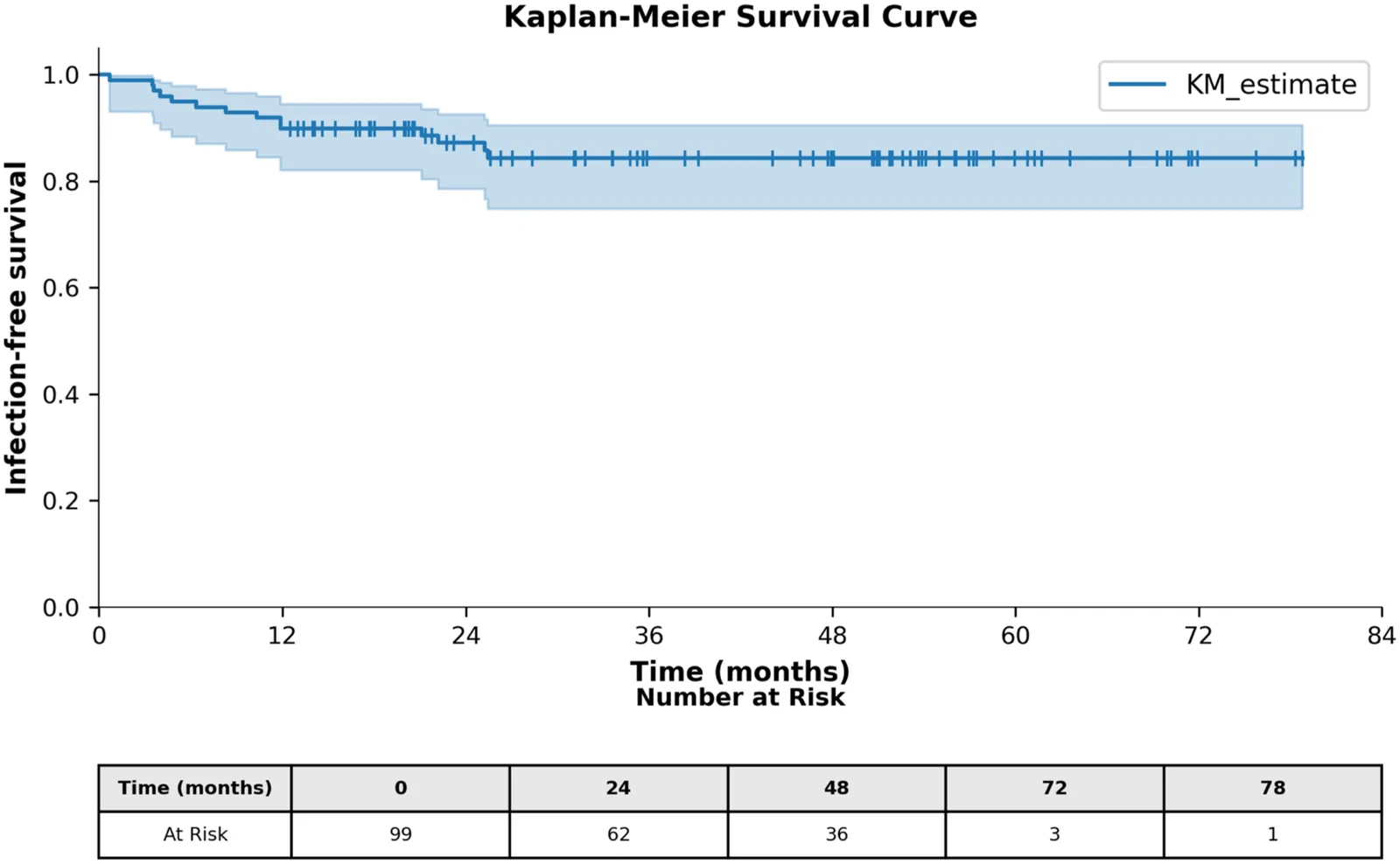
Kaplan–Meier curve of infection-free survival following guidewire exchange (*N* = 99). Infection-free survival rates at 12, 24, and 36 months were 93.7%, 87.3%, and 84.3%, respectively.

Technical success was achieved in all procedures (100%, 95% CI 96.3–100), and all catheters maintained an extracorporeal blood flow ⩾250 mL/min, ensuring adequate dialysis throughout follow-up.

## Discussion

This study demonstrates that the guidewire-exchange technique, performed using a protective stylet system, achieved low relapse and reinfection rates comparable to those observed with full catheter removal, with the added advantage of preserving the vascular access site. Relapse was conservatively defined as any recurrent bloodstream infection caused by the same pathogen as the index episode, irrespective of the time interval between events.

In our series of 99 tunneled CVC exchanges for CRBSI, relapse by the same pathogen occurred in only 2% of cases, and reinfection by different pathogens occurred in 11.1% of cases, with all catheters maintaining extracorporeal blood flow ⩾250 mL/min. Notably, although *Staphylococcus aureus* accounted for 73.2% of Gram-positive infections, only one relapse occurred with this pathogen. Because bacterial genomic typing was not available, it was not possible to determine whether these episodes represented true relapses or new infections.

Importantly, the long interval between the initial infection and the observed relapse by the same pathogens (3 and 8 months) suggests that these events may represent new infections rather than true relapses attributable to the guidewire-exchange procedure; under this interpretation, the relapse rate in our cohort would be close to zero. In addition, reinfections caused by different pathogens occurred after a median of 283 days, further indicating that these events are unlikely to be directly related to the guidewire-assisted exchange itself. Rather, these late infectious events likely reflect external factors, including variability in infections-control practices across dialysis centers and differences in adherence to hygiene protocols by nursing staff.

These results support the safety and efficacy of this approach in hemodialysis (HD) patients with limited vascular access. Our findings align with prior studies reporting comparable outcomes between guidewire exchange and catheter removal.^11–13^

A large multicenter analysis evaluating replacement strategies for tunneled HD catheters^14^ found no significant difference in catheter survival between over-the-guidewire exchange and new placement, although most infected catheters in that cohort were removed. Our study, which focuses specifically on infected CVCs, demonstrates that with an appropriate contamination-minimizing technique, guidewire exchange can be both feasible and effective. Recent data further support this conclusion. A pilot study describing a modified extra-catheter guidewire technique for in situ exchange of dysfunctional tunneled catheters^15^ reported favorable line patency and low complication rates.

From a mechanistic standpoint, the stylet-protected guidewire-exchange approach represents a significant technical innovation. In this study, we exclusively used Glidepath™ tunneled hemodialysis catheters placed in an anterograde fashion, which are structurally designed with an internal stylet intended to provide additional rigidity and control during catheter placement. Our technique exploits the geometric design of these antegrade tunneled Glidepath™ catheters by advancing the guidewire entirely within the hollow lumen of the internal stylet, rather than directly within the catheter lumen. By leveraging this intrinsic structural feature, the potentially contaminated guidewire remains physically separated from the inner surface of the new catheter throughout advancement, thereby preventing direct contact with the sterile luminal surface and minimizing the risk of intraluminal cross-contamination.

Positive guidewire cultures in our cohort confirmed persistence of the original CRBSI pathogen, yet relapse remained low, underscoring the protective value of the technique. The Kaplan–Meier curve demonstrated excellent long-term durability of this approach.

We did not perform fibrin sheath disruption (FSD), consistent with recent studies showing no significant relationship between FSD and infectious complications or infection-related mortality.^16,17^

Comorbidities such as diabetes and malnutrition, as reflected by hypoalbuminemia, as well as advanced age were frequent in our population and represent well-established risk factors for CRBSI. Hyperglycemia provides a favorable environment for bacterial proliferation, while age-related vascular fragility and immune senescence further increase susceptibility.^3^ Despite these risk factors, the technique demonstrated high success rates, supporting its reproducibility and clinical utility even in high-risk patients.

Clinically, this approach offers substantial advantages for interventional nephrologists. It preserves venous access, avoids morbidity associated with repeated venipunctures, and reduces the procedural burden associated with new catheter placement. Its simplicity and reproducibility make it well suited for integration into interventional nephrology training programs. The use of fluoroscopic guidance, meticulous aseptic technique, and proper tunneling remain essential for ensuring optimal outcomes.

## Limitations

This study has several limitations. First, its retrospective single-center design limits the generalizability of the findings and may introduce selection bias. Second, antibiotic management was heterogeneous: precluding a precise assessment of the impact of systemic antimicrobial therapy on outcomes. Nevertheless, the consistently high rates of infection-free survival observed suggest that the favorable results are more likely related to the guidewire-exchange technique itself rather than to antimicrobial therapy. Finally, bacterial genomic typing was not performed in the relapse cases; therefore, it was not possible to distinguish true relapses from new infections caused by genetically distinct strains.

## Conclusions

Central venous catheters remain a major vascular access modality for nearly one-third of hemodialysis patients, making their optimal management a cornerstone of nephrology care.

This study demonstrates that the stylet-protected guidewire-exchange technique is a safe, effective, and reproducible strategy for replacing infected tunneled CVCs. This approach preserves venous access, critical for the survival of HD patients, while providing favorable infection-free survival outcomes, even in cases caused by high-risk pathogens such as *Staphylococcus aureus*.

When performed according to the step-by-step technique described, this method minimizes contamination risk and ensures adequate catheter function. Prospective randomized clinical trials are warranted to further validate its role in the standard management of CRBSIs in hemodialysis patients.
